# NQO1 Binds and Supports SIRT1 Function

**DOI:** 10.3389/fphar.2021.671929

**Published:** 2021-06-21

**Authors:** Peter Tsvetkov, Julia Adler, Romano Strobelt, Yaarit Adamovich, Gad Asher, Nina Reuven, Yosef Shaul

**Affiliations:** ^1^ Broad Institute of Harvard and MIT, Cambridge, MA, United States; ^2^ Department of Molecular Genetics Weizmann Institute of Science, Rehovot, Israel

**Keywords:** quinone oxidoreductase 1, NADH/NAD ratio, SIRT1 activity, PGC1 alpha, mitochondria stress

## Abstract

Silent information regulator 2-related enzyme 1 (SIRT1) is an NAD^+^-dependent class III deacetylase and a key component of the cellular metabolic sensing pathway. The requirement of NAD^+^ for SIRT1 activity led us to assume that NQO1, an NADH oxidoreductase producing NAD^+^, regulates SIRT1 activity. We show here that SIRT1 is capable of increasing NQO1 (NAD(P)H Dehydrogenase Quinone 1) transcription and protein levels. NQO1 physically interacts with SIRT1 but not with an enzymatically dead SIRT1 H363Y mutant. The interaction of NQO1 with SIRT1 is markedly increased under mitochondrial inhibition. Interestingly, under this condition the nuclear pool of NQO1 is elevated. Depletion of NQO1 compromises the role of SIRT1 in inducing transcription of several target genes and eliminates the protective role of SIRT1 following mitochondrial inhibition. Our results suggest that SIRT1 and NQO1 form a regulatory loop where SIRT1 regulates NQO1 expression and NQO1 binds and mediates the protective role of SIRT1 during mitochondrial stress. The interplay between an NADH oxidoreductase enzyme and an NAD^+^ dependent deacetylase may act as a rheostat in sensing mitochondrial stress.

## Introduction

SIRT1 is a member of the NAD^+^ dependent class III histone/protein deacetylase family that is composed of seven members (SIRT1–7) that are evolutionarily conserved (Blander and Guarente, 2004). The Sirtuins are differentially localized in the cell. SIRT3, SIRT4, and SIRT5 are mitochondrial, SIRT2 is cytoplasmic whereas SIRT6, SIRT7, and SIRT1 are mainly nuclear (Haigis and Sinclair, 2010). SIRT1, the yeast Sir2 ortholog, is the most studied sirtuin family member. SIRT1 is active in the deacetylation of histones, transcription factors and enzymes involved in key cellular processes such as metabolism (Nemoto et al., 2005; Cantó et al., 2009), longevity (Kaeberlein et al., 1999), circadian oscillation (Asher et al., 2008) and differentiation (Fulco et al., 2003). Moreover, NAD^+^ plays a crucial role in activating SIRT1 activity. Under caloric restriction or fasting, the level of NAD^+^ rises and SIRT1 function is induced (Cantó et al., 2009; Canto et al.,2010; Hayashida et al., 2010). Increased levels of NAD^+^ due to elevated NAD^+^ salvage pathway synthesis, external NAD^+^ precursor addition or the inhibition of NAD^+^ utilizing enzymes such as PARP all have been shown to promote SIRT1 activity (Zhang et al., 2009; Bai et al., 2011; Li et al., 2016).

NQO1 is a ubiquitous flavoenzyme that catalyzes two-electron reduction of various quinones, utilizing NADH as an electron donor and generating NAD^+^ (Lind et al., 1990). In recent years it has become evident that NQO1 has a dual function in the cell. First, by associating with the 20S proteasome, it regulates the degradation of several intrinsically disordered proteins (IDPs) [as summarized in (Shaul et al., 2010)]. The second and more studied NQO1 role is as part of the cellular defense mechanism in response to electrophilic and/or oxidative stress generated by exposure to chemicals or endogenous quinones (Talalay and Dinkova-Kostova, 2004). NQO1 enzymatic activity shifts the cellular redox state as was shown in NQO1 knockout mice that exhibited a significant increase in the NADH/NAD^+^ ratio (Gaikwad et al., 2001). This is also the case in the involvement of NQO1 in the plasma membrane electron transfer (PMET) system highly regulating the NADH/NAD^+^ ratio and cellular viability following different cellular stresses, including mitochondrial stress (Haefeli et al., 2011).

NQO1 interacts with and activates SIRT2 in an NAD-dependent manner for completion of mitosis (Kang et al., 2018). NQO1 may supplement levels of NAD^+^ in supporting SIRT2 deacetylase activity (Siegel et al., 2021). Previously we proposed that NQO1 by catalyzing the oxidation of NADH to NAD^+^ might regulate SIRT1 activity (Asher et al., 2001). NQO1 regulates the protein levels of transcription co-activator PGC-1α, a key metabolic gene regulator (Adamovich et al., 2013). The stabilizing effect of NQO1 on PGC-1α is NADH-dependent and regulates PGC-1α accumulation and transcriptional activity following cellular starvation. PGC-1α is also activated by deacetylation by SIRT1 that in turn is regulated by the alteration in the cellular NADH/NAD^+^ ratio (Cantó et al., 2009). These findings and the ability of NQO1 to alter the NADH/NAD^+^ ratio in cells led us to explore a possible cross talk between SIRT1 and NQO1. Here we report on a functional physical interaction between NQO1 and SIRT1. We further demonstrate that NQO1 positively regulates SIRT1 activity in response to mitochondrial stress.

## Materials and Methods

### Cells and Cell Culture

293 human kidney cells (HEK293), HCT116 and NIH3T3 cells were grown in Dulbecco’s modified Eagle’s medium (DMEM) supplemented with 10% fetal bovine serum (FBS), 100 units/ml penicillin, and 100 mg/ml streptomycin and cultured at 37°C in a humidified incubator with 5.6% CO_2_. C2C12 cells were grown as previously described (Adamovich et al., 2013). H1299 cells with a YFP insertion in NQO1 exon 2 (clone 130207p11G9) was obtained from the laboratory collection of Uri Alon (http://www.weizmann.ac.il/mcb/UriAlon/DynamProt/index.html). All the cell lines were tested for *mycoplasma* contamination and found negative.

### Compounds

Resveratrol, rotenone, antimycin A, oligomycin, nicotinamide and cycloheximide were purchased from Sigma. EX527 was from PeproTech.

### Plasmids, Transfection and Retroviral Infection

The plasmids used were: pEFIRES NQO1, NQO1 C609T, flag-NQO1, flag-p73β. pEFIRES flag-SIRT1 and flag-SIRT1 H363Y, pcDNA3.1 flag-SIRT2, pcDNA3.1 flag-SIRT3 (provided by Eric Verdin), pBabe SIRT1 and pBabe SIRT1 H363Y (provided by Robert Weinberg), pcDNA3.1 flag-SIRT4-7 (provided by Haim Cohen) and the pCDNA3 Peredox-mCherry reporter (provided by Gary Yellen). Transient transfections of HEK293 cells were carried out by the calcium phosphate method. For retroviral infection HEK293T cells were transfected with pBabe GFP, pBabe SIRT1 or SIRT1 H363Y mutant together with Ψ-helper plasmid. Two days after transfection the supernatants containing the viral particles were harvested and used to infect NIH3T3 and C2C12 cells. NQO1 knockdown was achieved in C2C12 by introducing pLKO.1 shRNA against NQO1 (Sigma) and non-targeting control shRNA (Sigma) with lentivirus transduction. Lentiviral particles were produced in HEK293T cells according to the manufacturer's protocol.

### Immunoblot Analysis

Cell extracts and immunoblot analysis were carried out as previously described (Tsvetkov et al., 2011). The antibodies used were: goat anti NQO1 C19 and R20 (Santa Cruz), rabbit anti SIRT1 H300 (Santa Cruz) and mouse monoclonal anti His (Sigma).

### Co-Immunoprecipitation Studies

Co-immunoprecipitation experiments were carried out as previously described (Tsvetkov et al., 2011). Briefly, extracts from cells transiently transfected with indicated plasmids were incubated with Flag beads (Sigma), washed, eluted and separated on a 12.5% SDS-PAGE. *In vitro* co-immunoprecipitation experiment with recombinant purified His-SIRT1 and NQO1 was carried out with Ni-NTA beads (Qiagen) in buffer containing 50 mM Tris-HCl pH 8.0, 150 mM NaCl and 20 mM imidazole. Following washes samples were eluted in the same buffer with 200 mM imidazole. For nuclear fractionation cells’ pellet was 3 times washed and resuspended in nuclear extraction buffer containing 50 mM Tris-HCl pH = 7.5, 1% NP-40, 0.25% DOC, 150 mM NaCl, 1 mM EDTA. After 10 min incubation on ice the suspension was centrifuged for 15 min at 16,000 g, and the supernatant was collected as the nuclear fraction.

### Purification of Recombinant Proteins

pET28-His-TEV-NQO1 and pET28-His-SIRT1 were expressed in bacteria and cells were lyzed by sonication in 50 mM Tris-HCl pH 7.5, 150 mM NaCl, Lysozyme and 1 mM PMSF. Soluble His-TEV-NQO1 and His-SIRRT1 were purified using Ni-NTA column (HiTrap chelating HP) followed by gel filtration chromatography (HiLoad 16/60 superdex 200). Purified His-TEV-NQO1 was further cleaved by TEV protease and the His-TEV was removed upon binding to Ni-NTA column. For co-immunoprecipitation of endogenous SIRT and NQO1, mouse livers RIPA extracts were immunoprecipitated with either control protein A/G beads (Santa Cruz) or A/G beads coated with either non-immune rabbit IgG, or rabbit anti SIRT1 H300 (Santa Cruz). Protein levels were analyzed by Western blotting.

### Immunofluorescence Analysis

Cells were fixed with 4% paraformaldehyde, permeabilized with 0.5% Triton-X 100 and blocked with fetal calf serum containing 10% (v/v) skim milk. Cells were then incubated with goat anti NQO1 C19 and R20 antibodies (Santa Cruz). Following incubation with Cy5-conjugated donkey anti-goat antibody (Jackson Immuno Reasearch Laboratories), coverslips were mounted in DAPI Fluoromount-G (SouthernBiotech, Birmingham, AL, United States ). Microscopic images were obtained using laser scanning microscope LSM710 (Carl Zeiss, Microimaging GmbH, Göttingen, Germany) with plan-apochromat 63×/1.40 oil DIC M27 objective, and managed by Laser Sharp 2000 software (Zeiss, Munich, Germany). Representative images with identical laser intensities.

### Activity Assay

Cells were plated in 96 well plates and treated as described in the figure legend. NQO1 activity was assayed as reported (Prochaska and Santamaria, 1988).

### Viability Assay

Cell viability was analyzed using the XTT assay (Biological Industries) and spectrophotometrically quantified according to manufacturer’s instructions.

### Pulse-Chase Experiment

Pulse-chase experiments were conducted as described previously with slight modifications (Tsvetkov et al., 2009). HEK293T cells were incubated with medium depleted of methionine (−M) for 20 min [^35^S]Methionine (10 mCi/ml; Amersham Biosciences) was added reaching a final concentration of 0.2 mCi/μl for a pulse of 30 min. Cells were washed, and medium containing 2% unlabeled methionine was added for the indicated time (chase). Cells were collected, and protein extracts were subjected to immunoprecipitation as described above.

### Gene Expression Analysis

Total RNA was isolated from cells by using Tri reagent (Molecular Research Center), and reverse transcribed using the iScript cDNA kit (Bio-Rad). Gene expression levels were normalized to the geometric mean of three housekeeping genes, TATA binding protein (TBP), hypoxanthine phosphoribosyltransferase (HPRT), and glyceraldehyde-3-phosphate dehydrogenase (GAPDH) and expressed relative to control using the threshold cycle (ΔΔCT) method (Adamovich et al., 2013). The sequences of the primers used in this study will be provided upon request.

## Results

### Quinone Oxidoreductase 1 Binds SIRT1

**FIGURE 1:**
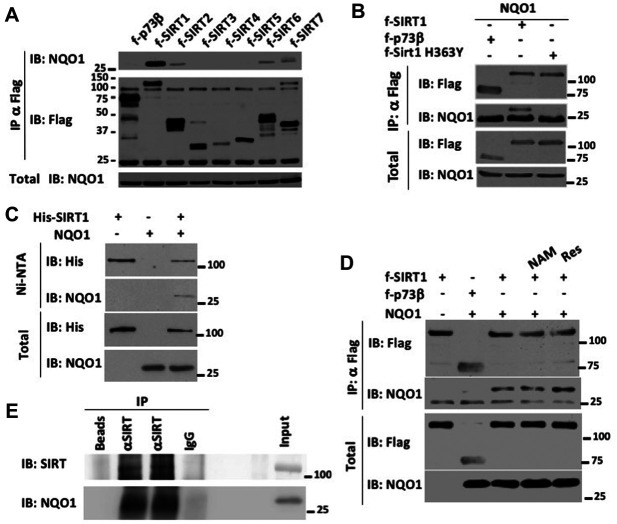
SIRT1 directly binds NQO1. **(A)** HEK293 cells were co-transfected with NQO1 and flag tagged sirtuins (SIRT1-7) or p73β (as negative control in all the transfection experiments). Following immunoprecipitation with flag beads the association of NQO1 was analyzed by immunoblot with the indicated antibodies. **(B)** Flag tagged SIRT1 or the SIRT1 H363Y mutant were co transfected in the presence of NQO1. The association of NQO1 was analyzed following immunoprecipitation with flag beads. **(C)** Bacterially expressed and purified His-SIRT1 and NQO1 were incubated and the binding of NQO1 was analyzed following precipitation of His-Sirt1 with Ni-NTA beads. **(D)** HEK293 over expressing flag tagged SIRT1 and NQO1 were treated with 10 mM NAM or 50 μM Resveratrol for 16 h and the association of NQO1 was examined following immunoprecipitation with flag beads. **(E)** Endogenous SIRT1 and NQO1 interact. Mouse liver extracts were immunoprecipitated (IP) with control empty beads, control IgG-coated beads, or anti-SIRT1-coated beads; and protein levels were analyzed by Western blotting (IB). The results of two anti-SIRT1 IPs are shown.

To investigate NQO1 interaction with the Sirtuin family members we performed co-immunoprecipitation assays. Flag SIRT1-7 and p73β as a negative control were transfected in the presence of NQO1 in HEK293 cells. The Sirtuins were immunoprecipitated with flag beads and the levels of co-immunoprecipitated NQO1 was analyzed. NQO1 showed high association with SIRT1 (Figure 1A). Mild association of NQO1 was also observed with SIRT2, SIRT6 and SIRT7. Next we asked whether SIRT1 functional conformation is required for NQO1 binding. SIRT1 H363Y is a catalytically inactive form of SIRT1 (Vaquero et al., 2007). The SIRT1 mutant did not bind NQO1 (Figure 1B) suggesting a functional conformation of SIRT1 is required for the interaction with NQO1. To further validate the direct physical association between SIRT1 and NQO1 we bacterially expressed and purified both proteins. NQO1 was incubated alone or in the presence of His-SIRT1 followed by precipitation with Ni-beads. NQO1 precipitated only in the presence of His-SIRT1 (Figure 1C) suggesting the binding between SIRT1 and NQO1 is direct.

Nicotinamide (NAM), the byproduct of SIRT1 enzymatic activity, has been shown to inhibit SIRT1 activity whereas the natural polyphenol resveratrol has been shown to activate SIRT1 activity in cells (Howitz et al., 2003). To examine whether SIRT1 activity modulators affect the binding to NQO1 we over expressed flag-SIRT1 with NQO1 in HEK293 cells and examined the co immunoprecipitation of NQO1 to flag-SIRT1 following treatment with either 10 mM NAM or 50 μM Resveratrol. These treatments did not affect NQO1 SIRT1 association (Figure 1D). Next we used mouse liver extract to ask whether endogenous SIRT1 interacts with NQO1. SIRT1 was immunoprecipitated and the level of NQO1 was examined by immunoblotting with NQO1 specific antibody (Figure 1E). These data suggest that the over-expressed and the endogenous NQO1 directly interact with SIRT1.

### SIRT1 Supports Quinone Oxidoreductase 1 Expression -

**FIGURE 2:**
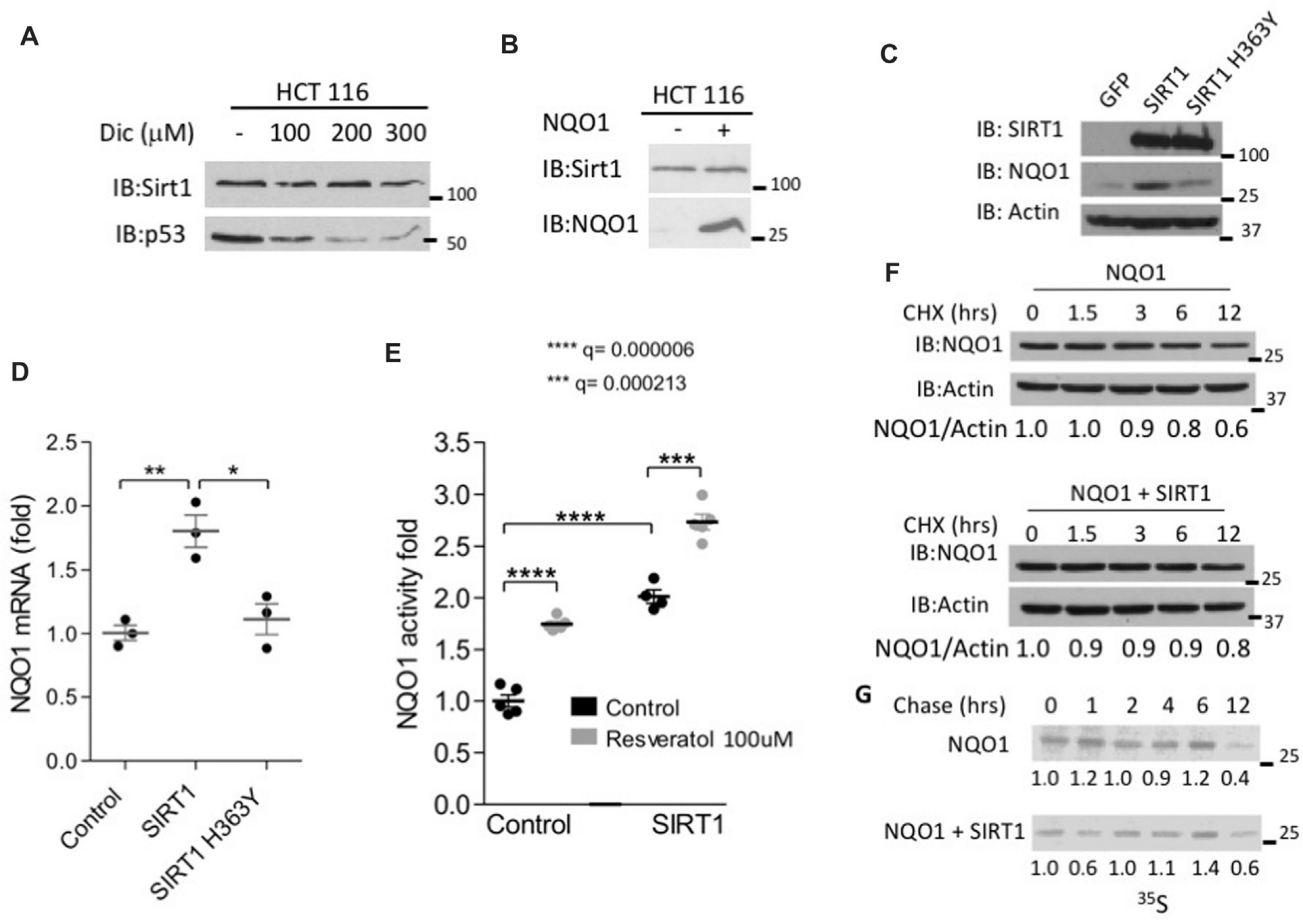
SIRT1 increases the endogenous NQO1 expression and protein level. **(A)** HCT116 cells were treated with indicated concentrations of dicoumarol and the level of SIRT1 and p53 were analyzed by Western blotting. **(B)** The protein level of SIRT1 was analyzed in HCT116 control and NQO1 overexpressing cells. NIH3T3 stably over expressing SIRT1, mutant H363Y SIRT1 or GFP as a control were examined for the NQO1 protein level **(C)**, mRNA level **(D)** and activity **(E)** in the presence or absence of Resveratrol as indicated, for 16 h. The stability of NQO1 was examined in HEK293 cells over expressing NQO1 in the presence or absence of flag-SIRT1 following cycloheximide treatment (100 μg/ml) at indicated time points **(F)** or following pulse labeling with ^35^S Methionine and harvesting at indicated time points following the addition of unlabeled methionine (chase)**(G)**.

The binding of NQO1 to SIRT1 suggests there is a functional interplay between the two enzymes. Given the role of NQO1 in protein degradation we examined the possibility that SIRT1 accumulation is regulated by NQO1. To this end we used dicoumarol, a potent inhibitor of NQO1, which promotes the degradation of NQO1-protected proteins, such as p53 (Asher et al., 2001). SIRT1 protein levels were not affected by increased doses of dicoumarol that induced p53 protein degradation (Figure 2A), Also NQO1 overexpression did not alter the level of the endogenous SIRT1 protein levels (Figure 2B). Thus, NQO1 binding to SIRT1 does not induce functional stabilization of SIRT1 but rather might be due to other functional interplay.

Next we explored the effect of SIRT1 on NQO1 levels. To this end we infected NIH3T3 cells with a control GFP or with either wild type or H363Y mutant SIRT1 expressing plasmids and followed the level and the activity of the endogenous NQO1. SIRT1 over-expression in this system increased NQO1 protein levels compared to the control GFP or the mutant H363Y SIRT1 expressing cells (Figure 2C). Similar results were obtained at the level of mRNA. In wild-type SIRT1 overexpressing cells, but not in the negative controls a 2-fold induction in NQO1 mRNA was observed (Figure 2D). Next, we explored the NQO1 enzymatic activity. Control and SIRT1 over expressing cells were treated with Resveratrol for 16 h. Following treatment, the cell extracts were analyzed for NQO1 enzymatic activity. Over expression of SIRT1 alone increased the level and the NQO1 activity by 2-fold whereas the addition of resveratrol further increased NQO1 activity by about 3-fold (Figure 2E), possibly via an increase at the level of NQO1 expression (Wu et al., 2006).

Over expression of SIRT1 did not alter the half-life of NQO1 as analyzed by inhibiting translation with cycloheximide (Figure 2F) or by ^35^S methionine pulse chase experiment (Figure 2G). Our data suggest that active SIRT1 up regulated NQO1 expression.

### Mitochondrial Inhibition Induced Quinone Oxidoreductase 1 Accumulation

**FIGURE 3:**
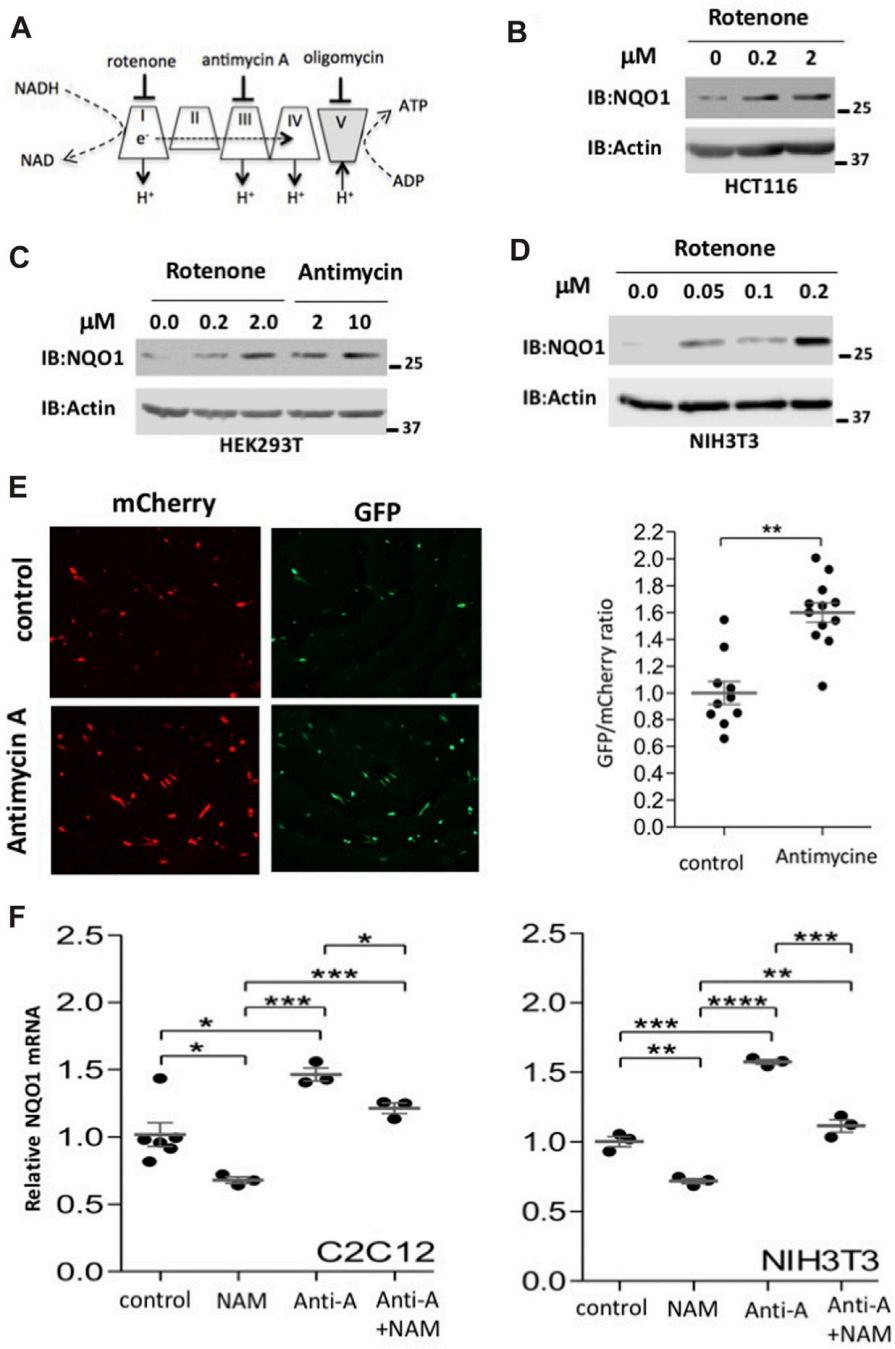
Mitochondrial inhibition induces NQO1 accumulation. **(A)** Mitochondrial respiratory complexes (I–V) and their specific inhibitors are depicted. **(B–D)**. The endogenous levels of NQO1 protein were examined in HCT116 **(B)**, HEK293T **(C)** and NIH3T3 **(D)** cells following treatment with indicated concentration of either rotenone (complex I inhibitor) or antimycin A (complex III inhibitor) for 16 h. **(E)** Using the pCDNA3 Peredox-mCherry reporter we measured changes at the level of the NADH/NAD^+^ ratio (higher GFP/mCherry means higher NADH/NAD^+^ ratio). Representative images and their quantifications are shown. **(F)** Inhibition of SIRT1 reduces NQO1 mRNA levels under basal and mitochondrial inhibition conditions. C2C12 or NIH3T3 cells were treated with antimycin A (10 μM) and NAM (10 mM) as indicated for 16 h and analyzed for NQO1 mRNA expression by quantitative PCR. The box-and-whisker plots show the results of t-test analyzed three independent experiments.

NQO1 is an oxidoreductase that reduces various quinones by oxidizing NADH to NAD^+^. During mitochondrial inhibition there is an increase in the NADH/NAD^+^ ratio and ROS, and both are expected to induce NQO1 activity. To inhibit mitochondrial oxidative respiration, we used rotenone to inhibit complex-I, or antimycin A to inhibit complex-III (Figure 3A). These inhibitors increase the NADH/NAD^+^ ratio. NQO1 protein levels markedly increased following mitochondrial inhibition. This behavior was not cell type specific and observed in HCT 116, HEK293T and NIH3T3 cells (Figures 3B–D).

Using the pCDNA3 Peredox-mCherry reporter (Hung et al., 2011) we show that the NADH/NAD^+^ ratio increases upon antimycin-A treatment as detected by higher GFP/mCherry ratio (Figure 3E). Next we tested the NQO1 RNA level and found that it is increased by antimycin A treatment both in C2C12 and NIH3T3 cells (Figure 3F). Interestingly, the NQO1 RNA levels are reduced by NAM, a known Sirtuins inhibitor. These results suggest that maximal induction of NQO1 expression under mitochondrial stress requires SIRT1 activity.

### Inhibition of the Mitochondria Increases Nuclear Quinone Oxidoreductase 1 Levels and NQO1-SIRT1 Association

**FIGURE 4:**
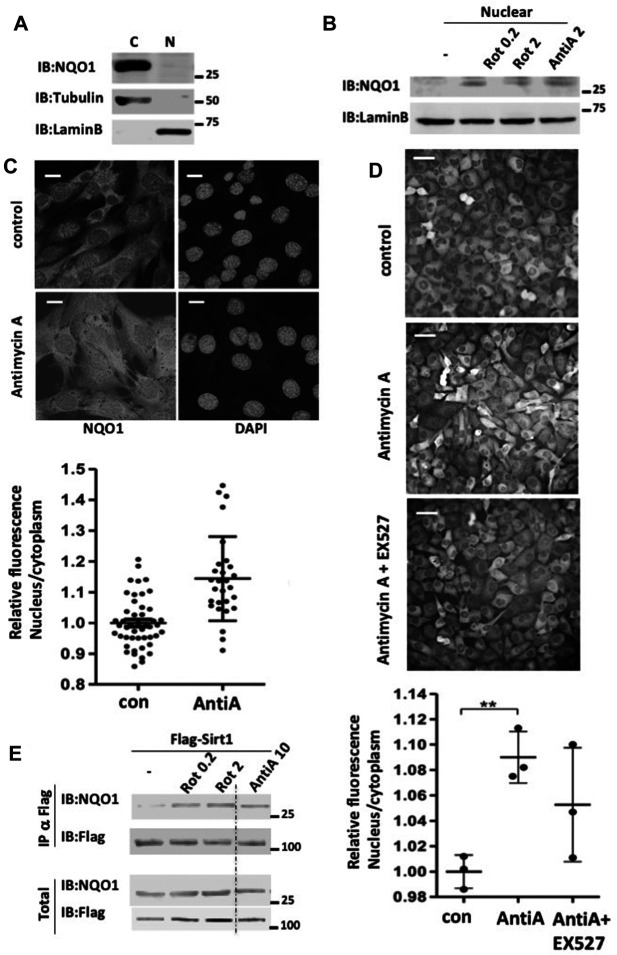
Mitochondrial inhibition increases nuclear NQO1 level and its SIRT1 association. **(A)** cytoplasmatic (C) and nuclear (N) NQO1distribution were analyzed in NIH3T3 cells. **(B)** The nuclear fraction of NQO1 was analyzed in NIH3T3 cells following treatment with indicated concentrations of rotenone or antimycin A for 16 h. **(C)** Subcellular distribution of NQO1 under conditions of mitochondrial inhibition. C2C12 cells were antimycin A treated (10 μM for 16 h) and fixed for immunofluorescence analysis with the NQO1 antibody (Epitomics 2618-1). Nuclear staining is indicated by DAPI. The quantification of the nuclear/cytoplasm ratio based on fluorescence intensity is shown (lower panel). Scale bar: 10 μm **(D)** H1299 cells in which the endogenous NQO1 gene contains YFP in the second exon (clone 130207p11G9) were antimycin A treated in the presence or absence of EX527 (25 μM), a specific Sirt1 inhibitor. The quantification of the nuclear/cytoplasm ratio based on fluorescence intensity measured of three independent field is shown (lower panel). Scale bar: 50 μm E) HEK293T cells over expressing Flag-SIRT1 and NQO1 were treated with indicated concentrations of rotenone or antimycin A for 16 h. Following the treatment the nuclear fraction was isolated and the association of NQO1 to SIRT1 in the nucleus was analyzed by flag immunoprecipitation.

It has been reported that NAD^+^ related metabolic enzymes are involved in regulating transcription (Zhang et al., 2009), and therefore we examined the cellular localization of NQO1. In untreated NIH3T3 cells the vast majority of the endogenous NQO1 was cytoplasmic (Figure 4A). Under mitochondrial inhibition NQO1 could be detected in the nucleus (Figure 4B). To further examine NQO1 nuclear localization under mitochondrial stress the endogenous NQO1 level in C2C12 cells was subjected to immunofluorescence analysis (Figure 4C). Antimycin A treatment increased NQO1 nuclear localization. Similar results, although to a lesser extent were obtained with H1299 cells in which the endogenous NQO1 gene contains YFP in the second exon (clone 130207p11G9). Interestingly, under this setting the NQO1 nuclear localization was partially reduced by EX527, a SIRT1 specific inhibitor (Figure 4D).

SIRT1 plays a protective role under mitochondrial inhibition conditions (Gerhart-Hines et al., 2007; Tanno et al., 2007). This led us to explore the possibility that the NQO1-SIRT1 interaction is improved under these conditions. Flag-SIRT1 was immuno-precipitated (IP) and the coIP of NQO1 was analyzed in the control HEK293 cells and cells that were treated with different concentrations of Rotenone and antimycin A. NQO1 coIP with SIRT1 markedly increased with both mitochondrial inhibitors (Figure 4E). These data suggest that during mitochondrial inhibition NQO1 is nuclear localized and that SIRT1-NQO1 physical interaction is increased.

### Quinone Oxidoreductase 1 Supports SIRT1 Regulated Gene Expression Under Mitochondrial Inhibition

**FIGURE 5:**
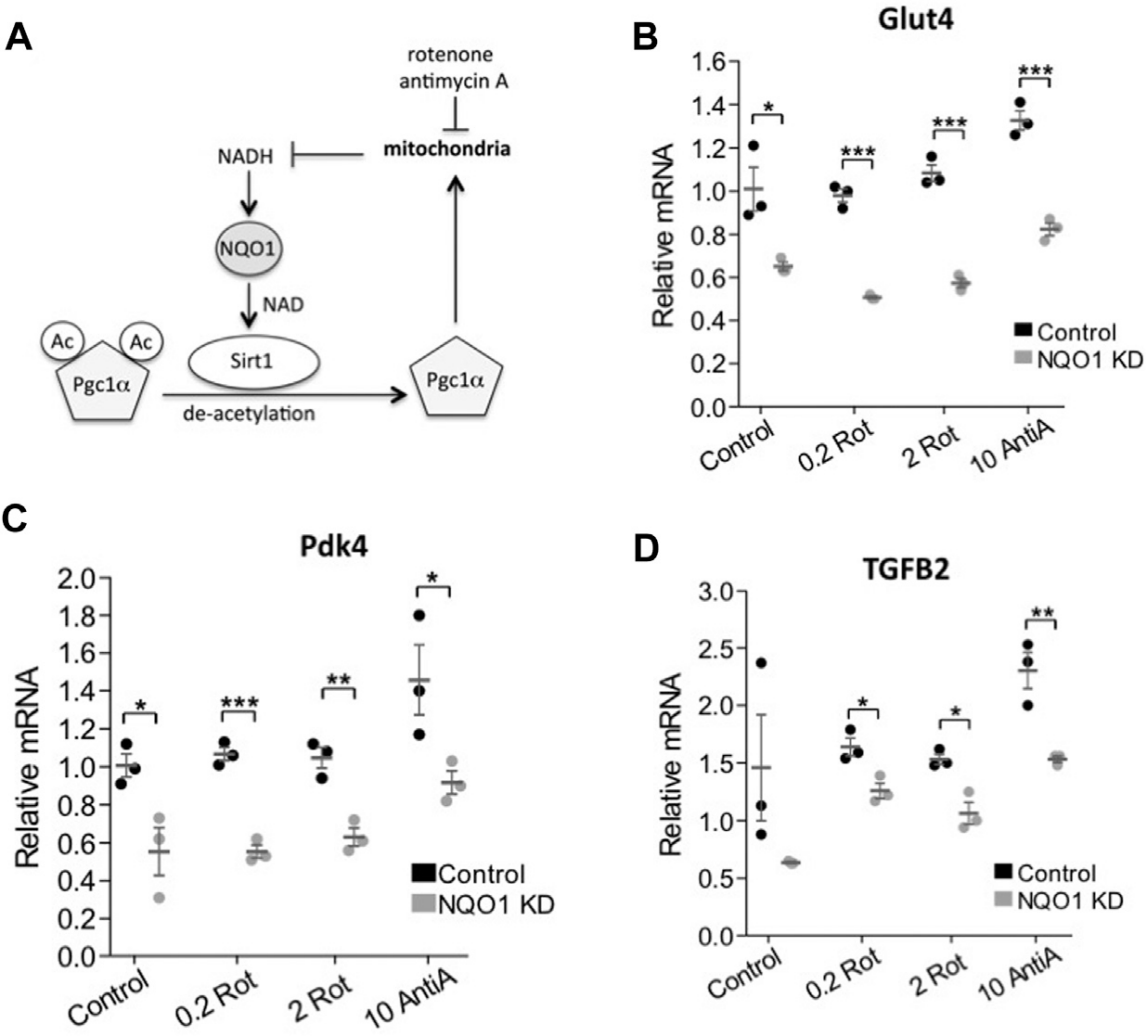
NQO1 supports SIRT1 regulated gene expression under mitochondrial inhibition. **(A)** The interplay and the reported mechanism of NQO1-SIRT1 and PGC-1α under mitochondrial stress are schematically presented. **(B–D)** The mRNA levels of the SIRT1 regulated genes Glut4 **(B)** Pdk4 **(C)** and TGFB2 **(D)** were examined in NIH3T3 cells stably over expressing an NQO1 targeting shRNA or a control shRNA following treatment with indicated concentrations of rotenone (rot) or antimycin A (AntiA) for 16 h. Statistical significance was analyzed by conducting two-tailed unpaired t-test. *p < 0.05, **p < 0.01, ***p < 0.001.

Next we wished to examine if NQO1 activity regulates SIRT1 function in transcription. We hypothesized that NQO1 stimulated SIRT1 deacetylase activity by converting NADH to the SIRT1 cofactor, NAD^+^. PGC-1α is a transcription coactivator involved in mitochondrial biogenesis (Wu et al., 1999) that is deacetylated by SIRT1, a process that potentiates PGC-1α activity. Interestingly, we have previously shown that NQO1 depletion increases PGC-1α acetylation (Adamovich et al., 2013) suggesting that NQO1 supports SIRT1 enzymatic function (Figure 5A).

The SIRT1-PGC-1α circuit induces the expression of pyruvate dehydrogenase kinase-4 (PDK4) and Glucose transporter type 4 (GLUT4) (Cantó et al., 2009). We next asked whether under mitochondrial stress NQO1 plays a role in regulating these genes. To this end we depleted NQO1 by the knockdown strategy (Adamovich et al., 2013). Interestingly, we found that under NQO1 depletion the expression of these genes was compromised (Figures 5B,C).

Nicotinamide phosphoribosyltransferase (NAMPT) and nicotinamide mononucleotide adenylyltransferase 1 (NMNAT-1) salvage pathway regulates NAD level and SIRT1 activity. One of the cellular genes highly affected by depletion of any of these enzymes is the transforming growth factor beta 2 (TGFB2) gene (Zhang et al., 2009). We examined the role of NQO1 in regulating SIRT1 mediated transcription of TGFB2. Similar to the role of NQO1 in the SIRT1-PGC-1α circuit, we found that NQO1 regulates the SIRT1-NAD salvage circuit as well (Figure 5D). These results suggest that NQO1 potentiates the transcription of the SIRT1 target genes in a more systemic fashion.

### NQO1-SIRT1 Circuit Determines Cell Fate

**FIGURE 6:**
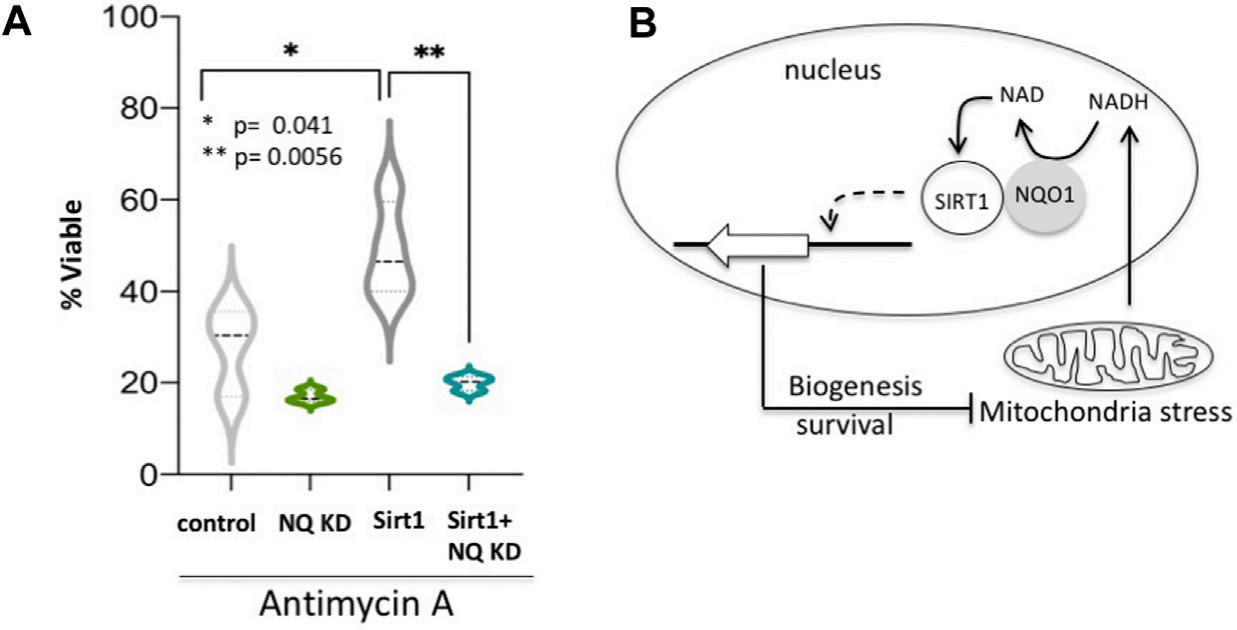
NQO1-SIRT1 circuit determines cell fate. **(A)** C2C12 cells either over expressing SIRT1 or the shRNA for NQO1 (NQ KD) or both were treated with Antimycin A and the viability was examined after 48 h. **(B)** The NADH metabolism in regulating the SIRT1-NQO1-PGC-1α feedback loop and mitochondrial biogenesis are schematically summarized.

Having revealed the NQO1-SIRT1 circuit in regulating gene expression under mitochondrial stresses we next investigated the role of this circuit in cell fate determination. It has been reported that nuclear SIRT1 protects C2C12 cells from antimycin A-induced cell death (Tanno et al., 2007). We asked whether NQO1 regulates the SIRT1-mediated protective role. We found that NQO1-depleted C2C12 cells are less viable under antimycin-A treatment (Figure 6A), whereas over-expression of SIRT1 increased cellular viability as previously described. Remarkably, the role of SIRT1 in maintaining viability is markedly blunted in NQO1-depleted cells. These data suggest that under mitochondrial stress NQO1 positively regulates nuclear SIRT1 in cell fate determination (Figure 6B).

## Discussion

In this work we functionally coupled NQO1 with SIRT1. We show that NQO1 physically interacts with the enzymatically active SIRT1. The NADH/NAD^+^ ratio in the cells is a rheostatic process whose set-point is dictated mainly by the mitochondria. NQO1 activity increases NAD^+^, the SIRT1 cofactor, coupling these enzymes in a functional module. We also provide evidence for a positive feed-forward loop whereby SIRT1 up-regulates NQO1 level. The observed interplay between NQO1 and SIRT1 establishes a new regulatory circuit sensing the NADH/NAD^+^ ratio. This circuitry is important in particular under conditions where the NADH/NAD^+^ levels are high such as mitochondrial stress. Under NQO1 depletion the ability of SIRT1 to maintain cell viability following mitochondrial inhibition is compromised. Our work suggests that the NQO1-SIRT1 regulatory circuit is an important component in cell fate decision-making in response to mitochondrial stress.

The regulatory circuit under study is activated by mitochondrial stress, conditions whereby PGC-1α is activated. Recently we have shown that NQO1 increases PGC-1α protein level in an NADH-dependent manner. The accumulated PGC-1α induces the expression of metabolic and mitochondrial biogenesis genes (Adamovich et al., 2013). We have now expanded this circuit by demonstrating that not only PGC-1α but also SIRT1 is regulated by NQO1. The ability of NQO1 to alter the NADH/NAD^+^ ratio attributes a central role to NQO1 in this regulatory PGC-1α/-SIRT1-NQO1 module.

Our co-immunoprecipitation data suggest that NQO1 physically interacts not only with SIRT1 but also with SIRT2, SIRT6 and SIRT7. All except SIRT1 are nuclear proteins whether NQO1 preferentially interacts with the nuclear SIRT family members is an interesting possibility demanding further investigations. However, NQO1 association with the cytoplasmic SIRT2 was also lately observed. The NQO1-SIRT2 interaction regulates mitotic progression (Kang et al., 2018) and acetylation of microtubules and α-tubulin (Siegel et al., 2021). In the case of mitotic progression, it was proposed that NQO1 functions by directly modulating NAD^+^ levels the cofactor of SIRT2 activity in a manner proposed here for SIRT1 activation.

The role of NQO1 described here expands the moonlighting functions of NQO1. NQO1 functions as a quinone reductase, one of the cellular phase-two detoxifying enzymes (Lind et al., 1990). However, NQO1 is also involved in transcription regulation, translation, posttranslational modifications and protein degradation (Asher et al., 2005; Adler et al., 2010; Alard et al., 2010; Siegel et al., 2021). NQO1 is associated with the 20S proteasome (Asher et al., 2005) and regulates the stability of numerous intrinsically disordered proteins (IDPs) such as p53, p73, and PGC1α in an NADH-dependent manner (Asher et al., 2005; Adamovich et al., 2013). This moonlighting behavior of a “metabolic enzyme” is not a unique property of NQO1. The PKM2 and GAPDH metabolic proteins are involved in numerous cellular processes. GAPDH for instance was shown to be involved in transcription regulation (Zheng et al., 2003) and as a chaperone of newly synthesized ribosomal protein L13a (Jia et al., 2012). To some extent these GAPDH activities are also NADH-dependent.

SIRT1 functions have been highly explored in the context of cellular aging and increased yeast life span (Kaeberlein et al., 1999). In the mouse model SIRT1 activity is beneficial in preventing pathologies associated with aging in tissues like muscle, brain and heart (Haigis and Sinclair, 2010). Although SIRT1 protein levels are high in some aged tissues its activity however remains highly dependent on the NAD^+^/NADH ratio (Alard et al., 2010; Tsvetkov et al., 2011). We have reported that NQO1 protein level significantly increase in the aging brain (Tsvetkov et al., 2011). Moreover, increasing the NADH/NAD^+^ ratio by facilitation of NQO1 activity is sufficient to delay muscle and brain functional decline in mice (Lee et al., 2012). These processes have been described to involve SIRT1, attributing an important role to the NQO1-SIRT1 circuit in aging.

In yeast the NQO1 homologue NQR1 directly increases both chronological and replicative lifespan. The replicative life span extension is induced by shifting cells toward respiratory metabolism, a process mediated by the SIRT1 yeast homologue SIR2 (Jiménez-Hidalgo et al., 2009). Thus, the functional link between the NADH oxidoreductases NQO1 (NQR1) and SIRT1 (SIR2) is evolutionarily conserved linking cellular metabolism to epigenetic regulation.

NADH/NAD^+^ levels and ratio are emerging as a critical cellular redox/metabolic sensing ratio regulating epigenetic, transcription, protein degradation and cellular metabolism (Ying, 2008). In the NQO1 knockout mouse one of the most significant effects reported is the alteration in the NADH/NAD^+^ ratio (Gaikwad et al., 2001), signifying the role of NQO1 in maintaining the cellular NADH/NAD^+^ levels. NQO1 ability to regulate the cellular NADH/NAD^+^ ratio is a key component of the plasma membrane redox system (PMRS) as well and highly affects cellular viability during oxidative and metabolic stresses (Tan and Berridge, 2010). However, the mechanism by which NQO1 mediates these functions is not yet well understood. We believe that the crosstalk between SIRT1 and NQO1 could directly link the NQO1 redox function with the cellular regulatory one.

NAD^+^ hydrolysis is an essential step in the SIRT1 de-acetylation reaction. One molecule of NAD^+^ is consumed and one molecule of o-acetyl-ADP ribose and nicotinamide is produced for every lysine de-acetylation reaction (Blander and Guarente, 2004). This NAD^+^ dependence links cellular metabolism with SIRT1 function in cells and suggests that physiological conditions that alter intracellular NAD^+^ levels or metabolic enzymes involved in NADH/NAD^+^ metabolism may regulate SIRT1 deacetylase activity, and the biological outcome. This has been shown to be true in yeast where mutation of the NPT1 gene encoding a nicotinate phosphoribosyltransferase, a critical enzyme in the NAD^+^ salvage pathway reduces NAD^+^ level and inhibits Sir2 mediated silencing (Sandmeier et al., 2002). In mammalian cells the NAD^+^ salvage pathway enzymes NMNAT-1 and NAMPT both increase NAD^+^ levels and activate SIRT1-mediated transcription regulation of a number of genes, including TGFB2 (Zhang et al., 2009). We show here that at least in the case of TGFB2 its transcription activation by SIRT1 is partially compromised under NQO1 depletion. Interestingly, PGC-1α itself has been reported to induce NAD biosynthesis (Tran et al., 2016). NAD^+^ has a central role in energy metabolism and is rate-limiting for mitochondrial function (Bai et al., 2011; Gomes et al., 2013). Therefore, NQO1, SIRT1 and PGC-1α are the core components of a metabolic rheostatic module responding to mitochondrial stress by sensing the NAD^+^/NADH ratio.

## Data Availability

The original contributions presented in the study are included in the article/Supplementary Material, further inquiries can be directed to the corresponding authors.
